# Detection of margin positivity in breast cancer using fluorescence and diffuse reflectance imaging

**DOI:** 10.1016/j.jtumed.2026.02.006

**Published:** 2026-03-02

**Authors:** Ankan Basu, Vijayendra Kedage, Subhash Narayanan, Phebe George, Rinoy Suvarnadas, Ajay Raveendranadh, Stanley Mathew

**Affiliations:** a Department of Surgery, Kasturba Medical College, Manipal Academy of Higher Education, Manipal, India; b Sascan Meditech Pvt Ltd, TIMed, Sree Chitra Tirunal Institute for Medical Sciences and Technology, Thiruvananthapuram, Kerala, India

**Keywords:** التصوير الفلوري, تصوير الانعكاس المنتشر, حواف الورم, الجراحة المحافظة على الثدي, Breast conservation surgery, Diffuse reflectance imaging, Fluorescence, Tumor margins

## Abstract

Accurate intraoperative assessment of surgical margins during breast-conserving surgery remains challenging due to tissue heterogeneity and the presence of small, scattered malignant foci. This prospective study evaluated the effectiveness of a multimodal imaging approach combining fluorescence imaging and diffuse reflectance spectroscopy (DRS) for detecting tumour involvement at surgical margins. Sixty female patients with a preoperative diagnosis of breast carcinoma were included. Immediately after surgical excision, multispectral images of resected specimens were acquired using a handheld imaging device. Measurements were obtained at wavelengths of 375 nm, 545 nm, 575 nm, and 610 nm and analyzed using proprietary software. Spectral readings from histologically confirmed malignant tissues were compared with those from histologically verified normal margins and benign lesions. A total of 510 multispectral images were obtained from 356 tissue locations, including 111 malignant lesions, 327 normal margins, and 72 benign lesions. The system demonstrated limited ability to differentiate benign from normal tissue (sensitivity 30.63%, specificity 63.96%). However, when distinguishing malignant from normal tissue at histologically confirmed margins (basal, inferior, lateral, medial, and superior), sensitivity ranged from 70.37% to 74.51%, while specificity ranged from 90.74% to 98%. Multimodal imaging using fluorescence and DRS demonstrates high specificity for detecting malignant tissue at surgical margins and shows promise as a practical, label-free intraoperative adjunct for margin assessment during breast-conserving surgery.

## الملخص

**أهداف البحث:**

أثناء إجراء الجراحة المحافظة على الثدي، يُعد تقييم وجود الأورام المتبقية على حواف تجويف الاستئصال أمرًا صعبًا بسبب طبيعتها غير المتجانسة ووجود بؤر صغيرة متناثرة من النسيج غير الطبيعي. أظهر استخدام التصوير الفلوري والتحليل الطيفي للانعكاس المنتشر قدرة كبيرة على كشف الأنسجة السرطانية. هدفت هذه الدراسة إلى تقييم كفاءة استخدام نهج تصوير متعدد الأنماط يجمع بين التصوير الفلوري والتحليل الطيفي للانعكاس المنتشر لتقييم حواف الورم لدى المرضى الخاضعين لجراحة سرطان الثدي.

**طرق البحث:**

أُجريت دراسة مستقبلية على 60 مريضة مشخصات مسبقًا بسرطان الثدي. تم الحصول على صور الفلورية والتحليل الطيفي للانعكاس المنتشر باستخدام كاميرا تصوير متعددة الأطياف محمولة باليد. التُقطت الصور متعددة الأطياف لعينة النسيج مباشرة بعد الاستئصال الجراحي للعينة. استُخدمت أطوال موجية قدرها 375 نانومتر، 545 نانومتر، 575 نانومتر، و610 نانومتر، وتم تحليلها باستخدام برنامج مخصص.

**النتائج:**

قورنت القراءات الطيفية للأنسجة الخبيثة المؤكدة نسيجيًا مع قراءات الأنسجة الطبيعية المؤكدة نسيجيًا المستخدمة كضوابط. تم تسجيل 510 صورة متعددة الأطياف من 356 موقعًا، منها 72 آفة حميدة، و327 حافة طبيعية، و111 آفة خبيثة. أظهرت النتائج حساسية قدرها 30.63% ونوعية قدرها 63.96% في التمييز بين النسيج الحميد والطبيعي. بينما أظهر التمييز بين النسيج الخبيث والنسيج الطبيعي عند الحواف القاعدية والسفلية والجانبية والإنسية والعلوية حساسية قدرها 70.37% و72.54% و74.51% و72.54% و74% على التوالي، ونوعية قدرها 90.74% و94.11% و94.11% و92.15% و98% على التوالي.

**الاستنتاجات:**

أظهر التصوير متعدد الأنماط الذي يجمع بين التصوير الفلوري والتحليل الطيفي للانعكاس المنتشر نوعية مرتفعة في كشف النسيج الخبيث في حواف الاستئصال الجراحي للثدي، وقد يمثل وسيلة عملية إضافية غير معتمدة على الوسوم للاستخدام أثناء العملية الجراحية لتقييم الحواف في الجراحة المحافظة على الثدي.

## Introduction

Breast cancer remains a global health priority, accounting for approximately 2.3 million new cases and 666,000 deaths worldwide in 2022.^1^ Achieving negative surgical margins is a critical determinant of oncologic and cosmetic outcomes in the multidisciplinary management of the disease. Despite advances in surgical techniques, intraoperative margin assessment continues to pose significant challenges, with re-excision rates ranging from 15 % to 35 %.^2^ These repeat procedures increase healthcare costs and are associated with higher mastectomy rates and suboptimal cosmetic results following breast-conserving surgery.

Frozen-section histopathological examination remains the reference standard for intraoperative margin assessment. However, this approach is time-consuming and labor-intensive, and places a considerable burden on pathology services, limiting its routine use in many centers.^3^ Consequently, there is a clear clinical need for rapid, non-destructive, and objective intraoperative techniques that are capable of accurately distinguishing malignant tissue from healthy tissue at the surgical margin.

Optical imaging modalities, including fluorescence imaging and diffuse reflectance spectroscopy (DRS), have emerged as promising tools for real-time tissue characterization. These techniques provide information about tumor-associated biochemical and structural changes without the need for destructive tissue processing or exogenous agents.^4^, ^5^, ^6^ Emerging evidence suggests that multimodal optical approaches based on integrating complementary information from fluorescence and DRS may improve the diagnostic performance compared with unimodal methods, and better support intraoperative surgical decision making.^7^, ^8^, ^9^, ^10^, ^11^, ^12^

In this study, we evaluated a cost-effective multimodal imaging system based on integrating fluorescence imaging and DRS for real-time tumor margin status assessment in breast cancer. The diagnostic performance of this system was assessed by comparing bimodal imaging findings with gold-standard histopathological examination in order to validate a practical, label-free approach for intraoperative margin evaluation and reduce the need for secondary surgical interventions.

## Materials and Methods

This prospective study was conducted at the Department of General Surgery, Kasturba Medical College and Hospital, Manipal, Karnataka, India, between October 2020 and December 2022. The study commenced after obtaining Institutional Review Board approval (652/2020).

All adult female patients with a pre-operative diagnosis of breast carcinoma and undergoing either wide local excision (WLE) with axillary lymph node dissection (ALND) or modified radical mastectomy (MRM) were included. The study also included adult female patients who underwent neoadjuvant chemotherapy/neoadjuvant hormonal therapy for breast carcinoma. In order to compare benign cases with malignant lesions, we recruited adult female patients with benign breast lesions.

### Device specifications

**Figure 1:**
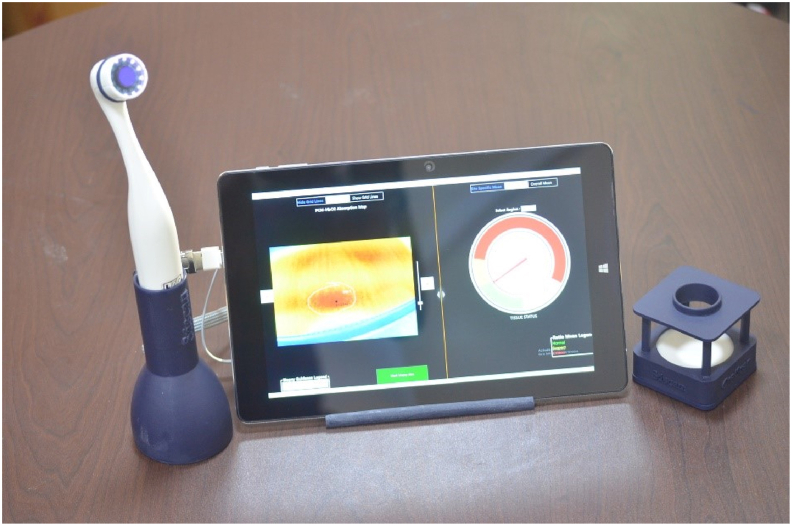
Handheld multispectral imaging device connected to the USB port of a Windows tablet and the calibration unit with a tissue phantom.

The handheld bimodal multispectral imaging system was developed by Sascan Meditech Pvt. Ltd for imaging epithelial tissues (Figure 1). The system consisted of a bimodal multispectral imaging device and an attached Windows tablet device with proprietary software preinstalled. This device has since been CE certified as a class I medical device (certification date: January 10, 2023; certificate no: CE-3866) for oral cancer screening, detection, and biopsy guidance. The device contained light-emitting diodes (LEDs) emitting at ultraviolet-1A (375 nm), green (545 nm), yellow (575 nm), and red (610 nm) wavelengths arranged in a circular array around a monochrome camera to illuminate the tissue. The light that emitted at 375 nm was useful for exciting collagen fluorescence. The wavelengths of 545 nm and 575 nm correspond to the absorption peaks for oxygenated hemoglobin (HbO_2_), and 610 nm corresponds to the wavelength at which deoxyhemoglobin (Hb) has a strong absorption cross-section compared with HbO_2_. The light collection optics used a long-pass filter that blocked 375 nm, and transmitted collagen fluorescence and elastically back-scattered light at 542, 577 nm, and 610 nm onto the camera's sensor. The novel optical device used crossed polarizers in the light-collecting and illumination pathways to remove specular reflection from the tissue's surface. The camera was connected to a Windows 10 (64-bit) Tablet, with proprietary software installed to control the sequential triggering of LEDs, image capture, and image processing. The device was calibrated to ambient room light conditions by using a tissue phantom before initiating the measurements.

The camera was focused on each tissue sample to capture multispectral images, i.e., Im 375, Im 545, Im 575, and Im 610. These images and the pseudo color-mapped (PCM) fluorescence image were processed using proprietary software. The PCM fluorescence image F375 and DRS ratio image R_610_/R_545_ were displayed on a screen.

### Methodology

After obtaining consent, this study included 60 female patients who were scheduled for either WLE with ALND or MRM and had preoperative core needle biopsy confirmation by histopathology that indicated breast cancer and other conditions mandating surgical excisions.

Each specimen was resected and retrieved, and provided to the study team in the operating theatre. To eliminate blood and debris, each specimen was cleaned with water under a running tap. The specimen was then oriented by taking numerous digital color photos with appropriate standardized labeling for identification and future use.

DRS and fluorescence images were captured from each edge of the excised breast specimen. If a residual breast cavity was present, the device also captured DRS and fluorescence images of the five edges of the cavity. It was necessary to ensure sterility when imaging the breast cavity, so images were acquired after covering the device with sterile transparent film. The operating team also removed a few representative lymph nodes and recorded their images. The removed lymph nodes were labeled individually. The entire recording process required around 10–20 min. The device was used within the operating room by switching off the room lights for cavity recording and an anteroom was utilized for recording specimens.

After recording the images, the investigator marked the regions of interest (RoIs) in the processed DRS images with a digital pen or mouse pointer by referencing the recorded color image. The margins of the specimen were marked as normal and lesions as abnormal. After marking a RoI, the software calculated the highest DRS ratio value in the marked RoI. This DRS ratio value was later correlated with the histopathology results for the specimen. Higher ratio values corresponded to higher tissue abnormalities and lower ratio values were representative of normal tissues.

The specimens were then fixed in formalin and transferred to the pathologist in the laboratory using the established protocol, before further histopathological analysis.

### Statistical analysis

The DRS ratio (R_610_/R_545_) values for different tissue types were correlated with the histopathology results, and statistical tests were conducted to assess the utility and accuracy of this approach for distinguishing between malignant, benign, and normal breast tissues using scatter plot diagrams. The sensitivity, specificity, negative predictive value (NPV), and positive predictive value (PPV) were determined.

A higher PPV indicated a high likelihood of correctly identifying positive cases, whereas a higher NPV denoted a high likelihood of correctly identifying negative cases.

## Results

Analysis of the age distribution showed that 45 % (27/60) of patients were aged less than 50 years, 41.7 % (25/60) were aged between 50 and 70 years, and the remaining 13.3 % (8/60) were older than 70 years. About 60 % (36/60) of the patients had coexisting illnesses, while 81.7 % (49/60) were preoperatively diagnosed with malignant breast disease, and 18.3 % (11/60) had benign pathology. The patients usually underwent MRM (61.7 %), followed by lumpectomy (20 %) and WLE with ALND (15 %). There was only one case of microdochectomy and one isolated lymph node excision (1.67 % each). Among the 60 patients, 36 (60 %) received neoadjuvant chemotherapy. Table 1 lists the post-operative histopathological diagnoses for the 60 patients.

**Table 1:** Post-operative histopathological diagnoses for 60 patients.

Post-operative histopathological diagnosis (N = 60)	Frequency (n)	Percentage (%)
Invasive breast carcinoma	41	68.33
Residual invasive breast carcinoma	7	11.67
Giant fibroadenoma	5	8.33
No residual invasive breast carcinoma	2	3.33
Fibro-adenomatoid hyperplasia	1	1.67
Intraductal papilloma	1	1.67
T-cell lymphoma	1	1.67
Benign phyllodes	1	1.67
Fibrocystic disease	1	1.67

**Figure 2:**
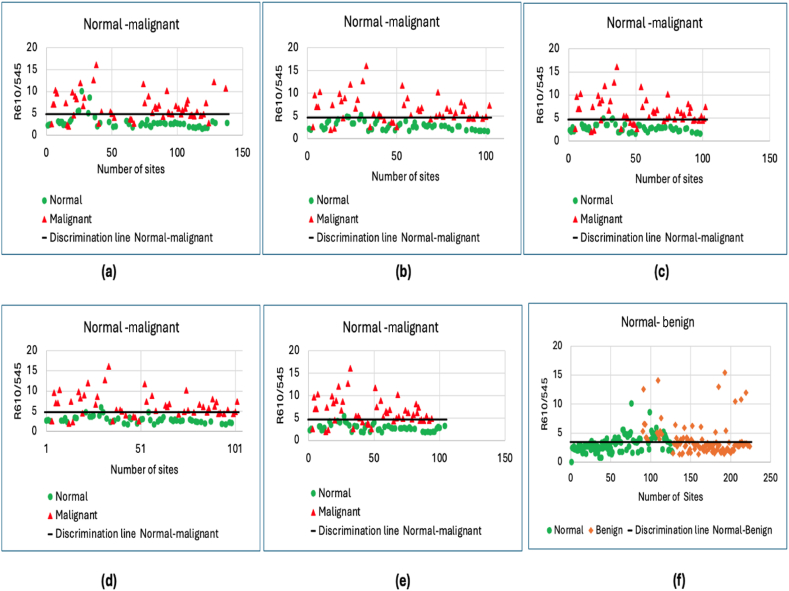
Scatter plots comparing R_610_/R_545_ ratio values between malignant cut-open specimens and normal tissues: (a) basal margin; (b) inferior margin; (c) lateral margin; (d) medial margin; (e) superior margin, and (f) benign versus normal tissue.

To assess the usefulness and accuracy of fluorescence and DRS for differentiating malignant, benign, and normal tissues in breast cancer patients, scatter plot diagrams were produced based on the DRS image ratios, R_610_/R_545_, for different sites (basal, inferior, lateral, medial, and superior margins). Cut-off lines were drawn at the average ratio values for the distinguishing sites. Figure 2 shows scatter plots for the R_610_/R_545_ ratios between malignant cut-open specimens and normal tissue at different sites, and between normal and benign tissues.

In total, 510 multispectral images were obtained from 356 sites, with 72 from benign lesions, 327 from normal margins, and 111 from malignant lesions. Assessment of the capacity to differentiate between benign and normal tissues yielded a sensitivity of 30.63 % and specificity of 63.96 %. However, the performance was higher for differentiating malignant versus normal tissue across basal, inferior, lateral, medial, and superior margins, with sensitivities of 70.37 %, 72.54 %, 74.51 %, 72.54 %, and 74 %, respectively, and specificities of 90.74 %, 94.11 %, 94.11 %, 92.15 %, and 98 %.

The predictive capacity of the technique for identifying positive tumor margins was analyzed by determining the sensitivity, specificity, PPV, and NPV for each site (Table 2).

**Table 2:** Confusion matrix comparing malignant tissue with normal margins.

Margin site	Sensitivity (%)	Specificity (%)	PPV (%)	NPV (%)	Mean malignant	Mean normal	Count malignant/Benign	Count normal
Malignant tissue vs. basal margin	70.37	90.74	88.37	75.38	6.6	2.94	54	54
Malignant tissue vs. inferior margin	72.55	94.11	92.5	77.42	6.49	2.83	51	51
Malignant tissue vs. lateral margin	74.5	94.1	92.68	78.68	6.49	2.75	51	51
Malignant tissue vs medial margin	72.54	92.15	90.24	77.04	6.49	2.92	51	51
Malignant tissue vs. superior margin	74	98	97.36	79.032	6.47	2.83	50	50
Benign tissue vs normal tissue	30.63	63.96	45.94	47.97	3.71	3.17	111	111

PPV: positive predictive value; NPV: negative predictive value.

**Figure 3:**
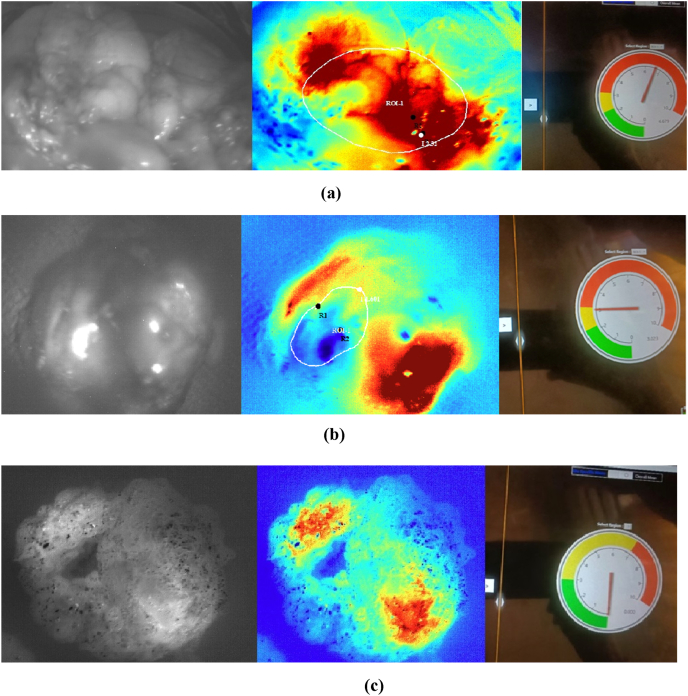
Monochromatic and PCM images captured by the multispectral imaging device: (a) malignant, (b) benign, and (c) normal tissues.

Spectral images of benign, malignant, and normal tissues captured using the device are shown in Figure 3.

## Discussion

Breast cancer continues to impose a substantial global health burden, contributing significantly to disability-adjusted life years and cancer-related mortality worldwide.^13^ Achieving negative surgical margins remains the objective of effective breast cancer management but intraoperative margin assessment is still challenging, often resulting in high re-excision rates. These challenges highlight the need for reliable, real-time intraoperative tools that can assist surgeons in margin evaluation, and potentially reduce the need for repeat surgeries.

Optical spectroscopy and imaging techniques have attracted increasing attention as intraoperative diagnostic tools due to their use of non-ionizing radiation, label-free operation, and ability to capture tumor-associated biochemical and structural alterations. Changes in cellular metabolism, stromal architecture, neovascularization, and tissue oxygenation generate distinct optical signatures that can be detected using fluorescence imaging and DRS. Autofluorescence from endogenous fluorophores such as NADH, FAD, and collagen reflects metabolic and extracellular matrix alterations, and DRS provides complementary information regarding hemoglobin absorption and tissue scattering associated with tumor angiogenesis.^14^^,^^15^

In the present study, integrating fluorescence imaging and DRS facilitated differentiation between malignant and normal breast tissue with high specificity across multiple margin orientations in an ex vivo setting. The observed diagnostic performance supports the concept that combining biochemical and structural optical information may enhance margin detection compared with unimodal approaches. The sensitivity was modest for differentiating benign from normal tissue, but this finding has limited clinical relevance because margin assessment is primarily critical in malignant disease. Importantly, the specificity was consistently higher for malignant tissues compared with histologically confirmed normal margins, highlighting the clinical utility of this multimodal approach for intraoperative margin assessment.

Previous studies obtained promising results using optical techniques for breast margin evaluation, where multimodal systems achieved sensitivities and specificities exceeding 90 % in controlled settings. However, many of these earlier approaches relied on complex spectral feature extraction, bulky fiber-optic probes, or exogenous contrast agents, thereby limiting their seamless integration into routine surgical workflows. By contrast, the imaging strategy evaluated in this study emphasizes real-time image-based assessment, workflow compatibility, and label-free operation, addressing key translational barriers identified in previous research.^16^^,^^17^ In particular, previous studies by Subhash et al. and Dao et al. established the importance of hemoglobin absorption bands at 542–577 nm and collagen fluorescence for tissue differentiation, and we translated these spectral signatures into a real-time, image-based ratio (R_610_/R_545_) that can be interpreted instantly in a surgical setting. Comparisons with fluorescence-guided systems using exogenous agents and radio frequency-based devices further highlight the trade-offs between sensitivity, specificity, and practicality across existing technologies.^18^, ^19^, ^20^, ^21^

Overall, the findings obtained in this study support the potential role of combined fluorescence and DRS imaging as a practical intraoperative adjunct for tumor margin delineation during breast-conserving surgery. Validation is required in larger, multi-center clinical studies to confirm the diagnostic performance of this method and to determine its impact on surgical decision making and re-excision rates.

## Strengths and limitations

A key strength of this study is the evaluation of a label-free, cost-effective multimodal imaging approach that integrates fluorescence imaging and DRS for real-time assessment of breast cancer margins. The system utilizes endogenous biochemical and structural tissue signatures, enabling high specificity when distinguishing malignant from normal tissue, without the need for exogenous fluorophores. In addition, image-based analysis and real-time processing enhance the workflow compatibility and support intraoperative decision making, addressing important translational barriers associated with previously proposed optical techniques.

However, several limitations should be acknowledged. The current probe design requires miniaturization and ergonomic optimization to allow easier access within the surgical cavity because it was originally developed for oral cavity applications. The operating-room lighting conditions also need to be standardized to minimize variability in image acquisition and ensure reproducibility. Furthermore, the cleaning protocol should be standardized to reduce the potential effects of surface artifacts on optical measurements. From a technological perspective, incorporating three-dimensional reconstruction of positive margins may further facilitate targeted re-excision. Future studies may also explore the application of this technique to assessing sentinel or regional lymph node involvement, potentially offering an adjunct to intraoperative frozen-section analysis in selected cases.

## Conclusion

The findings obtained in this study demonstrate that a label-free multimodal imaging system combining fluorescence and DRS provides a highly specific approach for real-time breast cancer margin assessment in an ex-vivo setting. By targeting endogenous metabolic and structural markers, the system has the ability to distinguish malignant from normal tissue without the need for exogenous agents. From a practical perspective, this multispectral imaging approach may be applied as a cost-effective intraoperative adjunct to conventional histopathology to support immediate surgical decision making. Future research should focus on hardware miniaturization to improve surgical cavity access, and validation in large-scale, multi-center clinical trials to assess the impacts on re-excision rates and long-term surgical outcomes.

## Ethics approval and consent to participate

Kasturba Medical College and Kasturba Hospital Institutional Ethics Committee – 652/2020. Ethics committee registration no: ECR/146/Inst/KA/2013/RR19. Participant recruitment commenced only after IRB approval and subsequent CTRI registration (CTRI/2021/01/030410).

## Consent for publication

Consent was obtained from all research participants before publication.

## Authors contributions

AB, VK, and SM conceptualized and designed the study, and collected and organized the data. AB, VK, SN, PG, RS, and SM analyzed and interpreted the data and prepared the manuscript. AR reviewed and edited the manuscript, and handled correspondence with the journal.

## Availability of data and material

The data that support this study's findings are available upon request from the corresponding author (AR). These data are not publicly available because they contain information that may compromise the privacy of research participants.

## Source of funding

This research did not receive any specific grant from funding agencies in the public, commercial, or not-for-profit sectors.

## Conflict of interest

The authors have no conflict of interest to declare.
